# The Molecular Composition of Dissolved Organic Matter in Forest Soils as a Function of pH and Temperature

**DOI:** 10.1371/journal.pone.0119188

**Published:** 2015-03-20

**Authors:** Vanessa-Nina Roth, Thorsten Dittmar, Reinhard Gaupp, Gerd Gleixner

**Affiliations:** 1 Max Planck Institute for Biogeochemistry, Jena, Germany; 2 Research Group for Marine Geochemistry (ICBM-MPI Bridging Group), University of Oldenburg, Institute for Chemistry and Biology of the Marine Environment (ICBM), Oldenburg, Germany; 3 Institute for Geosciences, Friedrich Schiller University, Jena, Germany; Old Dominion Univ., UNITED STATES

## Abstract

We examined the molecular composition of forest soil water during three different seasons at three different sites, using electrospray ionization Fourier transform ion cyclotron resonance mass spectrometry (ESI-FT-ICR-MS). We examined oxic soils and tested the hypothesis that pH and season correlate with the molecular composition of dissolved organic matter (DOM). We used molecular formulae and their relative intensity from ESI-FT-ICR-MS for statistical analysis. Applying unconstrained and constrained ordination methods, we observed that pH, dissolved organic carbon (DOC) concentration and season were the main factors correlating with DOM molecular composition. This result is consistent with a previous study where pH was a main driver of the molecular differences between DOM from oxic rivers and anoxic bog systems in the Yenisei River catchment. At a higher pH, the molecular formulae had a lower degree of unsaturation and oxygenation, lower molecular size and a higher abundance of nitrogen-containing compounds. These characteristics suggest a higher abundance of tannin connected to lower pH that possibly inhibited biological decomposition. Higher biological activity at a higher pH might also be related to the higher abundance of nitrogen-containing compounds. Comparing the seasons, we observed a decrease in unsaturation, molecular diversity and the number of nitrogen-containing compounds in the course of the year from March to November. Temperature possibly inhibited biological degradation during winter, which could cause the accumulation of a more diverse compound spectrum until the temperature increased again. Our findings suggest that the molecular composition of DOM in soil pore waters is dynamic and a function of ecosystem activity, pH and temperature.

## Introduction

Dissolved organic matter (DOM) is an important component of the global carbon cycle [1], [2]. The journey of terrestrial DOM from plants to the ocean starts when DOM is leached from decaying plant material or exuded from roots. Accompanied by sorption and desorption in the soil profile, DOM is transported to rivers and ultimately exported to the oceans [3–5]. Along this path, DOM is temporarily stored in the soils and aquatic systems, molecularly altered or mineralized to CO_2_. DOM is the main carbon and energy source for microorganisms in the aquatic food web [3], [6], [7]. After DOM reaches the ocean, the alteration continues and together with marine DOM, it provides the energetic basis for the microbial population in marine systems [8–11]. Via the microbial loop, DOM is not only respired but also transferred to higher trophic levels in the ocean [12], [13].

Changes in environmental conditions, e.g., in pH or temperature, may influence the degradation of DOM, leading to enhanced or reduced emissions of greenhouse gases into the atmosphere [14], [15]. In the case of DOM, a deep molecular-level understanding of its biogeochemistry is required to identify the key variables that influence its cycling [16–19].

Studies that examine DOM on the broad molecular level in relation to environmental factors are scarce. In the broader context of carbon storage and CO_2_ release into the atmosphere, more knowledge is needed about the processes affecting the origin and fate of DOM [20–22]. The photoreactivity of river DOM, fractionation of peat-derived DOM with metal salts and variations between DOM from bogs and fens demonstrate the relevance of environmental factors [23–26]. The molecular composition of DOM in headwaters, streams and lakes varies along climatic gradients and between biomes, but the key environmental parameters that control DOM molecular composition remain unknown [27], [28].

Recently, we [29] analyzed Yenisei tributary samples and bog samples from a 1000 km transect along the Yenisei River, Russia, using electrospray ionization Fourier transform ion cyclotron resonance mass spectrometry (ESI-FT-ICR-MS). This ultrahigh resolution technique ionizes a wide variety of dissolved organic compounds and detects molecule ions over a wide mass range [18], [30]. Because of the high resolution and mass accuracy, several thousand compounds and their molecular formulae can be identified in a sample [31–34]. Therefore, FT-ICR-MS is an appropriate method for studying DOM characteristics at the molecular level [19], [27], [28], [35–37]. In our Yenisei study, we revealed that pH and latitude were presumably the main drivers for the molecular-level variations of DOM, and we proposed their influence on biological processes that are involved in DOM processing. In the Yenisei transect study, however, we could not separate the effect of the highly correlated environmental factors, pH and DOC concentration, that both explained molecular differences in DOM from anoxic bogs and oxic rivers. Additionally, the effect of latitude was correlated with temperature. In the present study, we hypothesize that the correlation of pH and temperature with DOM molecular composition is universal and similar, e.g., in soil pore waters and the large Yenisei River. To test this hypothesis, we examined the molecular characteristics of DOM sampled at 5 cm depth from the mineral soil at three different forest sites in Germany that differ mainly in soil pH. To identify the effect of temperature, we analyzed samples from three different seasons. So far, studies examining the effect of environmental factors on the forest soil DOM composition were limited to bulk properties of DOM or a few target analytes [38–40]. By applying broadband molecular level-resolved analyses of several thousand individual DOM molecules, our approach provides new insights into the molecular characteristics.

## Material and Methods

### Sampling

Water samples were collected in March, May and November 2005 at three different long term monitoring sites (S1 Fig.) within a regular biweekly soil water sampling scheme. The first site is the well described [41–46] old growth beech forest in the Hainich National Park [51° 04’ 46” N, 10° 27’ 08” E, 440 m above sea level (a.s.l.)], Thuringia, central Germany. The unmanaged and deciduous forest is composed mainly of European beech (*Fagus sylvatica*, 65%) and ash (*Fraxinus excelsior*, 25%). The A horizon is 5 to 15 cm deep followed by a clayish T horizon on 50 to 60 cm deep fertile cambisol (clay loam). The experiments in the National Park were conducted with the permission of the National Park Administration.

The second site is the Wetzstein spruce forest (50° 27’ 13” N; 11° 27’ 27” E; 785 m a.s.l). The main tree species in the managed forest are ca. 50 yr old Norway spruce (*Picea abies*). The soil is acidic and carbonate-free [42], [43], [45]. The podzol (sandy loam) developed from carbonate-free parent material.

The third site is in a stand of maple trees (*Acer pseudoplatanus*), planted in 1990 within a pine and spruce forest (*Pinus sylvestris* and *Picea abies*) near the village of Thann in southeast Germany (49° 19’ 24” N, 12° 27’ 37” E). The granite parent material is followed by cambisol with high sand content and 5 to 7 cm organic layer on top [47]. No specific permissions were necessary to work on the Wetzstein and Thann site and no protected species were involved.

The temperature data were continuously logged at Wetzstein and Hainich (NTC resistance thermometer type 107, Campbell Scientific with radiation protection and data logger type CR23X, Campbell Scientific) and summarized to provide the daily average temperature. The length of the growing season was determined by summing the number of days with daily average temperature > 5°C. For the Thann site this was determined using data from Weiden, the nearest weather station available from ‘Deutscher Wetterdienst’ at http://www.dwd.de.

The water samples were taken using permanently installed glass ceramic suction plates (1–1.6 μm pore size) at 5 cm depth from the A horizon. We examined a total of 16 samples from three main sites in March, May and November 2005 (Table 1 and S1 Fig.). If only one sample was available from one designated spot per main site, we examined replicate measurements or used samples from the next deeper depth (10 cm).

**Table 1:** Overview of sample site, corresponding pH and DOC concentration, sampling period and length of growing season.

Site	Plot number designation	Sampling depth (cm)	Sampling month	Sample	pH	DOC (mg/L)	Sampling period in 2005	Length of the growing season (days)
Hainich	2	5	March	HS2–5-Mar	5.3	12.1	8–23 March	11
	4	5	March	HS4–5-Mar	5.5	9.2		
	2	5	May	HS2–5-May	5.0	17.4	5–3 May	55
	4	5	May	HS4–5-May	5.0	15.7		
	2	5	November	HS2–5-Nov	5.0	27.6	2–25 Nov	237
	4	5	November	HS4–5-Nov	5.0	20.9		
Thann	1	5	March	T-5-March	4.5	55.5	9–19 March	8
	1	5	May	T-5-May	4.5	89.7	4–25 May	70
	1	5	November	T-5-Nov	4.3	42.7	6–23 Dec	235
Wetzstein	1	5	March	W1–5-Mar	4.0	69.1	3–17 March	0
	3	10	March	W3–10-Mar	4.0	120.5		
	1	5	May	W1–5-May	4.3	96.0	11–26 May	41
	3	5	May	W3–5-May	4.3	94.4		
	3	10	May	W3–10-May	4.0	151.9		
	1	5	November	W1–5-Nov	4.0	61.9	26–6 Dec	199
	3	5	November	W3–5-Nov	4.0	100.1		

### Sample preparation and FT-ICR-MS analysis

After each sampling event, the bottles connected to the glass suction plates were evacuated to 20 kPa. Soil water was sucked into the bottle until ambient pressure was reached. Because of the biweekly sampling period for soil water, the DOM resulted from soil water collected over a period of two weeks (Table 1 and S2 Fig.). In the case of low sample volume, time points were combined. The samples were immediately freeze-dried in the lab and stored at room temperature in the dark. Prior to FT-ICR-MS analysis, the samples were redissolved in ultrapure water and desalted via solid phase extraction (Varian PPL; 1 g; [48]). The average extraction efficiency was 63% on a carbon basis. The DOC concentrate was diluted for FT-ICR-MS measurements with ultrapure water and methanol to 1:1 methanol/water (v/v) and a final concentration of 20 mg DOC/l. Acidified ultrapure water was stored in the same type of bottles as redissolved DOM samples and was used as a procedural blank for extraction and FT-ICR-MS measurements.

FT-ICR-MS measurements were performed at the University of Oldenburg (Germany). The samples were continuously infused into the ESI source at 120 μl/h and an ESI needle voltage of -4 kV. The FT-ICR-MS (Bruker Solarix, 15 Tesla) was used in negative ionization mode, and over *m/z* 150–2000 with an ion accumulation time of 0.25 s, 500 single scans were added to one spectrum. The internal calibration of the spectra was carried out with an in-house mass reference list.

### Statistical analysis

We only used *m/z* values with a signal/noise ratio (S/N) > 3 and within the range from *m/z* 150 to 800. Ions beyond *m/z* 800 were not detected in our samples. The m/z values detected in the procedural blanks were removed from the peak list of the samples. C, H, O, N and S (N ≤ 2, S ≤ 1; [24], [36]) were considered for molecular formula assignment with an in-house algorithm. Only singly charged ions occurred in our mass spectrum. In total, we assigned formulae to 5003 of the 7778 peaks. Most of the unassigned masses are isotopologues of the identified formulae. To compare samples and exclude the peaks that were not measured significantly in all samples, we defined a limit of detection (LOD) that was applied to all samples (LOD_Group_). For this purpose, the signals I_j,n_ of each measurement j with n peaks were normalized to the sum of the intensities Σ(I_j,n_) of each measurement resulting in normalized peak intensities I_j.n,Norm_ (I_j.n,Norm_ = I_j,n_/ Σ_n_(I_j,n_)). For each measurement the lowest I_j,n,Norm_ was set as an individual limit of detection (LOD_Ind_). LOD_Group_ was defined as the maximum of every LOD_Ind_ [29]. All peaks with a relative intensity < 2 * LOD_Group_ (limit of quantification) were set to 1.5 * LOD_Group_ to exclude variability between samples based on peaks near the LOD_Group_ [49]. Introducing LOD_Group_ resulted in a list of 1918 formulae for statistical analysis (S1 Table). These were based on 1062 to 1334 individual peaks per sample, of which 607 formulae were detected in all measurements from all samples. Therefore, 46 to 57% of all formulae per measurement occurred in all other measurements.

Peak reproducibility was determined with measurements of a deep sea DOM reference sample [50],[51] performed at the beginning and end of each of the two measurement days resulting into four replicate measurements. These measurements were handled as a separate data set with its individual LOD_Group_ resulting in 2045 to 2075 individual peaks with assigned molecular formulae for each measurement (S2 Table). According to [52], we determined a) the number of peaks shared between the four replicate measurements (1703), b) the proportion of peaks shared between the four replicates (83%); and c) the median of the relative standard deviation for the peak intensities of peaks found in all injections (15%).

For data interpretation, we only considered compounds with assigned molecular formulae because we wanted to extract information with respect to DOM chemical characteristics. Isotopologues do not provide additional molecular information and were also disregarded from further consideration. From the molecular formulae we calculated the following parameters: number of carbon (C#), hydrogen (H#), oxygen (O#) and nitrogen (N#) atoms, hydrogen to carbon ratio (H/C), oxygen to carbon ratio (O/C), double bond equivalents (DBE) and molecular weight (MW), double bond equivalents to carbon ratio (DBE/C), double bond equivalents to oxygen ratio (DBE/O), the difference between DBE and number of oxygen atoms (DBE-O), the aromaticity index, AI [53] and the nominal oxidation state of carbon (NOSC) [24]. Whereas H/C indicates the amount of hydrogen saturation, DBE indicate the number of π bonds and rings in a compound. Additionally, high DBE/C values indicate aromatic or condensed aromatic structures and the AI can be used to unambiguously identify aromatic (AI > 0.5) and condensed aromatic (AI ≥ 0.67) compounds [53]. DBE-O is a rough measure for C = C bonds because it omits any possible C = O bond [23]. O/C describes the degree of oxygenation [30]. Expressing the average oxidation state of all carbons in one formula, NOSC provides information on the biogeochemical reactivity of a compound [24]. Van Krevelen diagrams are a convenient and common method to display an overview over all H/C and O/C ratios of a data set [26], [54]. From the location in the van Krevelen diagram, a given formula can be roughly assigned to classes of compounds such as carbohydrates, lipid, peptides, lignin, and tannins [16], [30]. It must be emphasized, however, that the assignment of structural compound groups is ambiguous, with the exception of aromatic and condensed aromatic structures.

The statistical analysis was based on the data matrix containing normalized signal intensities and the corresponding molecular formulae. To find similarities and differences in the molecular data, we applied unconstrained and constrained ordination methods. Principal component analysis (PCA), as an unconstrained indirect gradient analysis, detects assumed gradients in the data set. Redundancy analysis (RDA), as a direct constrained gradient analysis, uses additional environmental information (Table 1) to explain the variance in the data set. The applicability of RDA, assuming a linear ordination model, was tested using detrended constrained canonical analysis (DCCA). The resulting length value of the longest gradient was < 1.3, rejecting the unimodal model and supporting suitability for the linear model [55]. The ordination methods were performed using Canoco 5 for Windows (Microcomputer Power, Ithaca, New York, United States). Matlab R2012a (The MathWorks, Inc., Natick, Massachusetts, United States) was used for further data preparation, data handling and statistical tests.

To test whether average values between different sites were significantly different, we applied Kruskal-Wallis followed by Mann-Whitney U tests. The Kruskal-Wallis test indicates whether at least one pair of parameters is significantly different from each other. The Mann-Whitney U test tests each pair separately. Both tests were performed using SPSS 16.0 for Windows (SPSS Inc., Chicago, Illinois, United States). The statistical significance was determined for all tests by applying a significance level of p < 0.05.

## Results

### Environmental data, pH and DOC concentration

The pH at the three sites ranged between 4.0 (minimum at Wetzstein) and 5.5 (maximum at Hainich, Table 1) and the mean pH values at the respective sites were 4.1 (± 0.1) at Wetzstein, 4.4 (± 0.1) at Thann, and 5.1 (± 0.2) at Hainich. Mann-Whitney U tests confirmed significantly different pH conditions between the sites. The DOC concentration ranged between 9.2 mg/l (minimum at Hainich) and 152 mg/l (maximum at Wetzstein) and the mean DOC concentrations were 99 (± 30) mg/L at Wetzstein, 63 (± 24) mg/l at Thann, and 17 (± 7) mg/l at Hainich. Mann-Whitney U tests confirmed significantly different DOC concentrations between Hainich compared to Thann and Wetzstein. Comparing the DOC concentrations between Thann and Wetzstein resulted into a p-value (p = 0.053) slightly above our significance level. We found a negative correlation between the pH and DOC concentration of the individual sample across all sites with a Spearman’s rank correlation coefficient, r_s_, of -0.87.

All three sites had a similar seasonal trend in temperature and a similar irregular precipitation distribution across the year (S2 Fig.). The samples were collected at comparable time points of the growing season in March, May and November (Table 1). The annual precipitation in 2005 was 735 mm at Hainich, 600 mm at Thann, and 580 mm at Wetzstein.

### Principal component and redundancy analysis (PCA and RDA)

**Fig 1:**
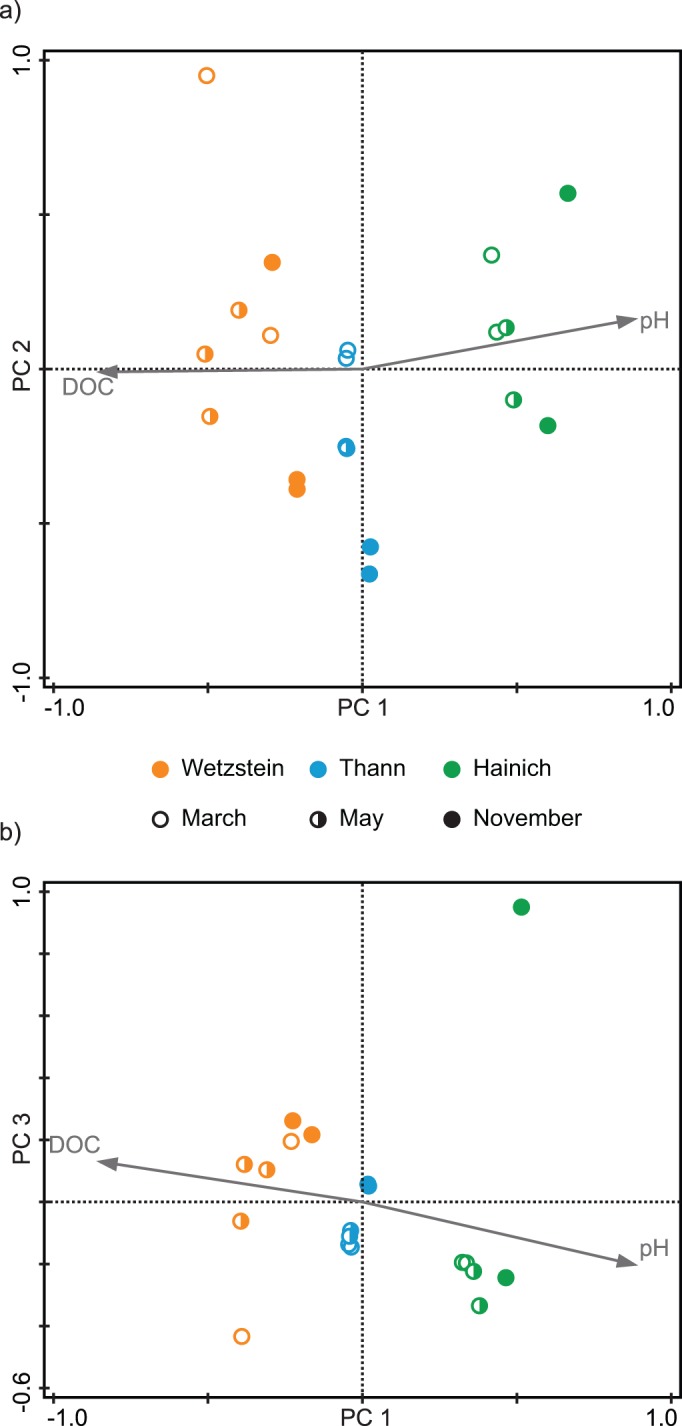
Ordination plots from PCA, based on all detected molecular formulae and their normalized FT-ICR-MS signal intensities. DOC concentration and pH were not used for PCA, but plotted as supplementary variables. Variability explained: PC1, 32.9%; PC2, 15.7%; PC3, 12.5%. (a) Plot of first and second axes, (b) plot of first and third axes.

PCA summarized 61% of the variability in the molecular data by the first three principal components (PC): PC1, 32.9%; PC2, 15.7%; PC3, 12.5%. PCA clearly separated the three sampling sites along axis 1 (Fig. 1). Both the pH and DOC concentration correlated with PC1. None of the known environmental parameters were correlated with PC2. PC3 appeared to be related to the sampling season. The majority of the samples collected in March had negative PC3 values, the May samples were more positive around the origin of the PC3 axis (Wetzstein and Thann) and most November samples had positive PC3 values. In summary, PCA revealed clear differences in DOM molecular composition. These differences were related to both the sampling site and season.

**Fig 2:**
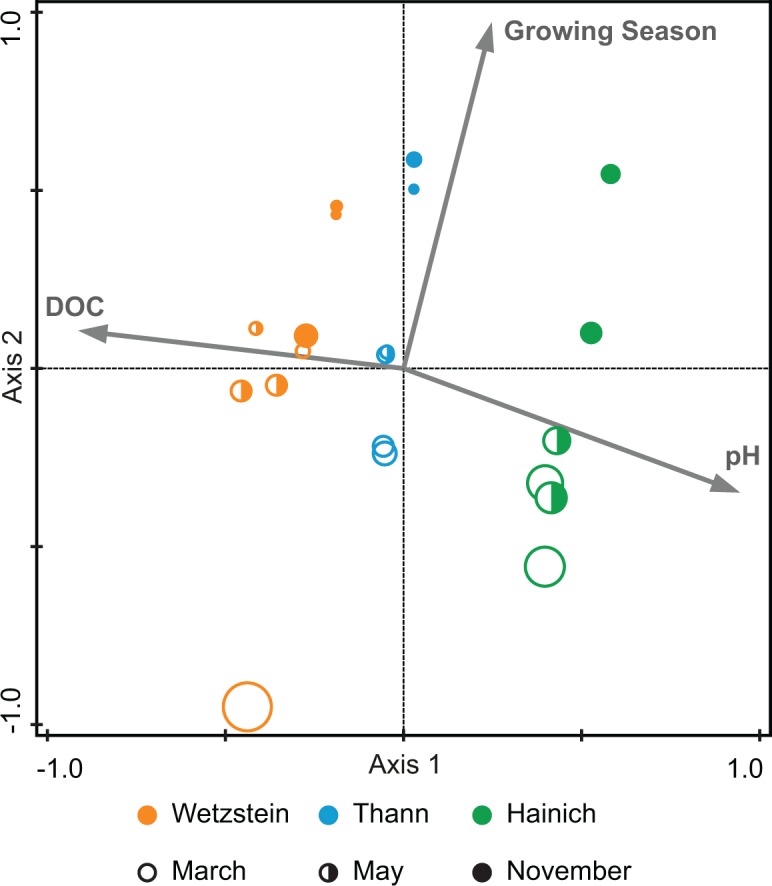
Species diversity diagram based on RDA with molecular formulae as responsive variables and pH, DOC concentration and length of growing season as explanatory variables. Variability explained: Axis 1, 30%; Axis 2, 8.6%. DOC concentration, pH and length of growing season together explain 42% of the variability in formulae. The greater the circle diameter, the greater the number of molecular formulae per measurement (range of formulae per measurement: 1062 to 1334).

The forward selection function in combination with Monte Carlo permutation tests applied in the RDA demonstrated that DOC concentration, pH and the length of the growing season explained 42% of the variability in the molecular data. Consistent with the PCA (Fig. 1), the first RDA axis separated the sampling sites and axis 2 the seasons. The variability along RDA axis 1 is correlated with DOC concentration and pH, the variability along axis 2 with season (Fig. 2). The molecular diversity (number of molecular formulae) decreased with the progression of the growing season from March to May and November. This indicated that the total number of formulae per measurement was related to temperature-related seasonal differences.

We applied variation partitioning with conditional effects implemented in Canoco 5 to test if pH and DOC concentration together explained variability independent from the length of the growing season. We grouped the pH and DOC concentration together because they were negatively correlated and displayed opposing effects in PCA and RDA (Fig. 1 and 2). Variation partitioning with conditional effects was based on three different RDAs. The first tested the total explained variation by pH, DOC concentration and length of growing season together. Because these were all the significant environmental factors, their explained variation of 42% was set to 100% of total explained variation. The second RDA tested how much of the total explained variation was explained by DOC concentration and pH together (84.9%), while the covariate role was assigned to length of growing season. The third RDA tested how much of the total explained variation was explained by the length of the growing season (21.6%), while the covariate role was assigned to pH and DOC concentration. The intersection c represented the amount of shared variability of both tested groups. To obtain this intersection, the unique parts were subtracted from 100%: c = (100–84.9–21.6)% = -6.5%. This slightly negative intersection can be regarded as not different from zero [56] and demonstrates that the DOC concentration and pH explain the variability independent of the length of growing season. The F statistics in Canoco 5 confirmed the significance of the fractions ‘pH and DOC concentration + length of the growing season + intersection’, ‘pH and DOC concentration’ and ‘length of growing season’.

To further examine the peaks correlating with the pH and DOC concentration, we applied variation partitioning to test the simple effects of each individual parameter by ignoring the other parameters. Again, this was based on three individual RDAs. The first RDA tested the total explained variation by pH and DOC concentration together (32.4%) and was set to 100% total explained variation. The second RDA tested how much of the total explained variation was explained by the DOC concentration (83.9%) without assigning a covariate role to pH. Resulting from that, 16.1% of the total explained variation (100%- 83.9%) was explained by pH. The third RDA tested how much of the total explained variation was explained by pH (93.4%) without assigning a covariate role to DOC concentration. DOC concentration explained 6.6% of the total explained variation (100%- 93.4%). Consequently, 77.3% (100%- 16.1%- 6.6%) of the total explained variation was explained by the shared effects of pH and DOC concentration. This high shared amount of explained variability was due to the negative correlation of pH and DOC concentration. Again, the F statistics in Canoco 5 confirmed the significance of the fraction ‘DOC concentration + intersection’, ‘pH + intersection’ and ‘DOC concentration + pH + intersection’. Here, we applied variation partitioning with simple effects because we intended to extract the molecular formulae that correlated both with pH and DOC concentration.

### Separating the effect of DOC concentration and pH

**Fig 3:**
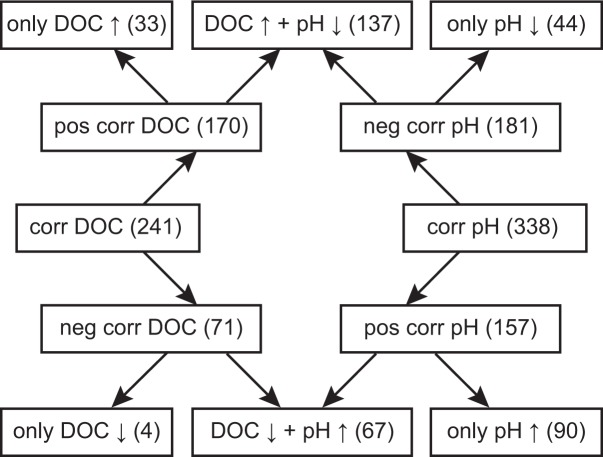
Overview of the different groups of molecular formulae that correlated significantly in different combinations with DOC concentration and pH. Numbers of molecular formulae in each group are in brackets.

Because RDA is based on regression analysis, we could also compute the coefficients of determination (R^2^) of every molecular formula with each environmental parameter. We used R^2^ from the partial RDAs executed in the variation partitioning to identify those molecular formulae that were significantly correlated with pH and DOC (R^2^ > 0.5). This resulted in two data sets: the formulae correlating with the DOC concentration (‘corr DOC’) and the formulae correlating with pH (‘corr pH’). Each of these data sets was separated into two further data sets. The formulae correlating with DOC concentration were separated into formulae correlating positively (‘pos corr DOC’) and negatively (‘neg corr DOC’) with DOC concentration. The same procedure was carried out for pH (Fig. 3). Because DOC concentration and pH were negatively correlated, they shared a certain amount of correlating formulae. These shared formulae were combined in the data set with formulae positively correlated with DOC and negatively with pH ‘DOC ↑ + pH ↓’ (S5 Table) and in the data set with those formulae negatively correlated with the DOC concentration and positively with pH ‘DOC ↓ + pH ↑’ (S6 Table). The remaining formulae were assigned to four other data sets of formulae: formulae that were correlated positively (‘only DOC ↑’) (S3 Table) or negatively (‘only DOC ↓’) (S4 Table) with DOC and those correlated positively (‘only pH ↑’) (S8 Table) or negatively (‘only pH ↓’) (S7 Table) with pH. Only 4 formulae were uniquely negatively correlated with DOC (‘only DOC ↓’), while 33 to 137 molecular formulae represented the other types of unique correlations (Fig. 3).

**Fig 4:**
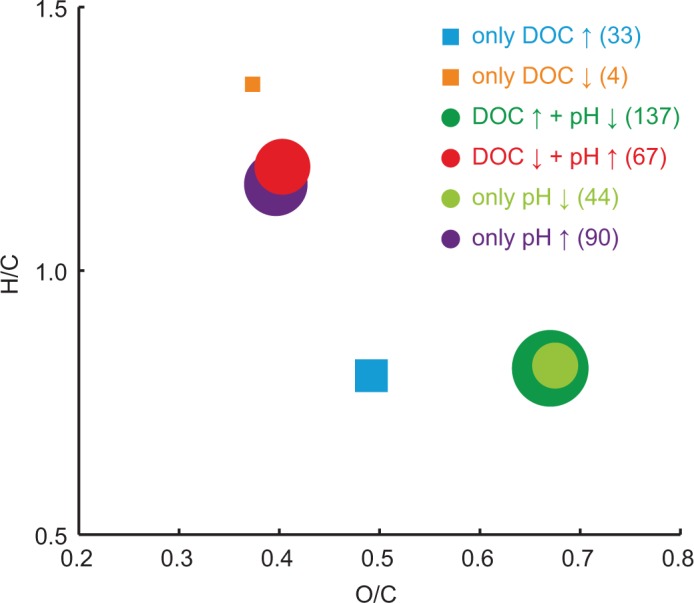
H/C and O/C data for the different groups of molecular formulae exhibited clear trends with pH and DOC concentration. The data for each group of formulae are summarized as centroid data in the van Krevelen diagram. The number of formulae per group is represented by the scaled size of the symbols and given in brackets in the legend.

An initial overview of the similarity and dissimilarity between the individual data sets of the extracted formulae is obtained by plotting their position in H/C versus O/C space (i.e., van Krevelen diagrams). To include the data for all six data sets in one figure and present it clearly, we reduced the information by plotting the centroids of each data set (Fig. 4). The trends found for the shared correlations (‘DOC ↑ + pH ↓’ and ‘DOC ↓ + pH ↑’) are similar to those for the unique correlation with pH (‘only pH ↑’ and ‘only pH ↓’) indicated by intersecting centroids. The formulae ‘only pH ↑’ and ‘DOC ↓ + pH ↑’ were characterized by higher H/C and lower O/C, whereas ‘only pH ↓’ and ‘DOC ↑ + pH ↓’ were characterized by lower H/C and higher O/C. Therefore, higher molecular saturation and less oxygenation were statistically related to higher pH and lower DOC concentration. The centroids of those formulae correlating uniquely with DOC concentration were only clearly separated from each other by H/C, i.e., higher unsaturation was related to higher DOC concentration. While ‘only DOC ↑’ was similar in H/C as ‘only pH ↓’ and ‘DOC ↑ + pH ↓’, it was separated by lower oxygenation. Consistently, ‘only DOC ↓’ was different from ‘DOC ↓ + pH ↑’ and ‘only pH ↑’ by H/C, while having a similar degree of oxygenation.

**Fig 5:**
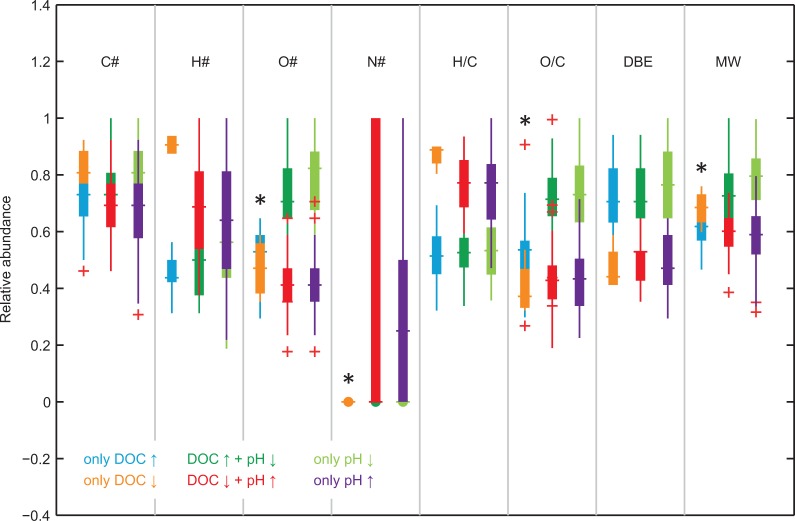
Boxplots representing the characteristics of the different groups of molecular formulae. The data are standardized to the maximum value of each parameter. Pairs with equal medians are marked *.

To compare the different data sets (Fig. 3) of the isolated molecular formulae in more detail, we used several parameters that are derived from the molecular formulae (C#, H#, O#, N#, H/C, O/C, DBE, MW, DBE/C, DBE/O, AI and DBE-O). We standardized the data for each of the parameters to the parameter maximum among all six data sets to display the boxplots for all parameters on one scale (Fig. 5, Table 2). Mann-Whitney U tests of the non-standardized data (‘only DOC ↑’ ↔ ‘only DOC ↓’, ‘only pH ↑’ ↔ ‘only pH ↓’, ‘DOC ↑ + pH ↓’ ↔ ‘DOC ↓ + pH ↑’) revealed statistically identical medians only for the data sets ‘only DOC ↑’ and ‘only DOC ↓’ concerning parameters O#, N#, O/C and MW. All other groups of molecules were significantly different from each other.

**Table 2:** Median, quartiles Q1 (25^th^ percentile) and Q3 (75^th^ percentile) and minimum and maximum values of various molecular parameters.

Negatively correlated with DOC and positively correlated with pH						Positively correlated with DOC and negatively correlated with pH					
	median	Q1	Q3	min	max		median	Q1	Q3	min	max
C	18.00	16.00	20.00	12.00	24.00	C	19.00	16.00	21.00	13.00	26.00
H	22.00	17.25	26.00	12.00	32.00	H	16.00	12.00	18.00	10.00	24.00
N	0.00	0.00	2.00	0.00	2.00	N	0.00	0.00	0.00	0.00	0.00
O	7.00	6.00	8.00	3.00	11.00	O	12.00	11.00	14.00	6.00	17.00
O/C	0.40	0.34	0.45	0.18	0.65	O/C	0.67	0.61	0.74	0.32	0.93
H/C	1.20	1.07	1.33	0.92	1.45	H/C	0.82	0.74	0.90	0.53	1.00
DBE	9.00	7.25	9.00	6.00	11.00	DBE	12.00	11.00	14.00	8.00	16.00
DBE/C	0.45	0.39	0.58	0.32	0.69	DBE/C	0.65	0.60	0.69	0.55	0.79
DBE/O	1.14	1.00	1.43	0.78	2.33	DBE/O	1.00	0.86	1.09	0.67	2.50
AI	0.08	0.00	0.21	0.00	0.50	AI	0.00	0.00	0.14	0.00	0.69
DBE-O	1.00	0.00	3.00	-2.00	5.00	DBE-O	0.00	-2.00	1.00	-5.00	9.00
MW	365.10	332.09	393.14	234.08	447.20	MW	441.03	391.06	489.04	309.03	607.09

Uniquely positively correlated with pH						Uniquely negatively correlated with pH					
	median	Q1	Q3	min	max		median	Q1	Q3	min	max
C	18.00	15.00	20.00	9.00	24.00	C	21.00	18.00	23.00	8.00	26.00
H	20.50	15.00	26.00	7.00	32.00	H	18.00	14.00	20.00	6.00	22.00
N	0.50	0.00	1.00	0.00	2.00	N	0.00	0.00	0.00	0.00	0.00
O	7.00	6.00	8.00	3.00	12.00	O	14.00	11.50	15.00	7.00	17.00
O/C	0.40	0.32	0.47	0.21	0.67	O/C	0.68	0.59	0.78	0.38	0.93
H/C	1.20	1.00	1.30	0.73	1.56	H/C	0.83	0.70	0.96	0.56	1.05
DBE	8.00	7.00	10.00	5.00	11.00	DBE	13.00	11.00	15.00	6.00	17.00
DBE/C	0.47	0.39	0.62	0.28	0.78	DBE/C	0.63	0.57	0.71	0.52	0.79
DBE/O	1.25	1.00	1.50	0.71	3.00	DBE/O	0.93	0.79	1.14	0.64	2.00
AI	0.14	0.00	0.28	0.00	0.63	AI	0.00	0.00	0.23	0.00	0.62
DBE-O	2.00	0.00	3.00	-3.00	6.00	DBE-O	-1.00	-3.00	2.00	-5.00	8.00
MW	358.12	315.16	397.15	192.03	483.19	MW	483.05	432.03	521.08	213.00	605.08

Uniquely negatively correlated with DOC						Uniquely positively correlated with DOC					
	median	Q1	Q3	min	max		median	Q1	Q3	min	max
C	21.00	20.00	23.00	20.00	24.00	C	19.00	17.00	20.00	12.00	22.00
H	29.00	28.00	30.00	28.00	30.00	H	14.00	13.50	16.00	10.00	18.00
N	0.00	0.00	0.00	0.00	0.00	N	0.00	0.00	0.00	0.00	0.00
O	8.00	6.50	9.50	6.00	10.00	O	9.00	8.00	10.00	5.00	11.00
O/C	0.35	0.31	0.44	0.30	0.50	O/C	0.50	0.42	0.53	0.25	0.85
H/C	1.38	1.31	1.40	1.25	1.40	H/C	0.80	0.70	0.91	0.50	1.08
DBE	7.50	7.00	9.00	7.00	10.00	DBE	12.00	10.75	14.00	7.00	16.00
DBE/C	0.36	0.35	0.39	0.35	0.42	DBE/C	0.65	0.60	0.70	0.54	0.80
DBE/O	1.13	0.91	1.15	0.70	1.17	DBE/O	1.38	1.18	1.57	0.64	2.80
AI	0.07	0.03	0.07	0.00	0.07	AI	0.36	0.19	0.45	0.00	0.69
DBE-O	1.00	-1.00	1.00	-3.00	1.00	DBE-O	3.00	1.75	5.00	-4.00	9.00
MW	416.18	384.19	444.17	363.18	461.18	MW	375.07	345.06	399.04	283.05	443.06

Data are shown for those molecular formulae that correlated negatively and positively with pH in the soil pore waters.

Consistent with the van Krevelen analysis (Fig. 4), the trends for ‘only pH ↑’ compared to ‘only pH ↓’ were very similar to the differences between ‘DOC ↓ + pH ↑’ and ‘DOC ↑ + pH ↓’. The trends demonstrated higher saturation (high H/C and lower DBE), lower oxygenation (O/C), smaller molecules (MW) and the presence of nitrogen-containing compounds at a higher pH and lower DOC concentration. The unique influence of pH (‘only pH ↑’ ↔ ‘only pH ↓’) was similar to the shared effect. Approximately 50% of the formulae that were related to high pH contained at least one nitrogen atom, about half of which even contained two nitrogen atoms (Fig. 5). Following this trend with pH, these nitrogen-containing compounds occurred mainly in the samples from the Hainich site (62 to 78), and rarely in the samples from Thann (2 to 7) and Wetzstein (0 to 8).

Because the medians of H#, H/C and DBE were higher in ‘only DOC ↓’ we conclude that the main difference between the two data sets of ‘only DOC ↑’ and ‘only DOC ↓’ was saturation. The unique effect of DOC (‘only DOC ↑’ ↔ ‘only DOC ↓’) was most obvious in the medians of H/C and H#. This was also indicated by the centroid data in the van Krevelen diagram (Fig. 4). Our results demonstrate that high DOC concentrations have a unique effect by increasing molecular unsaturation. The main trends in the unique effects of pH are similar to the shared effects of pH and DOC concentration. Overall, with higher pH and lower DOC concentration we found higher saturation, lower oxygenation, smaller molecules and abundant nitrogen-containing compounds.

### Seasonal differences in molecular characteristics

**Fig 6:**
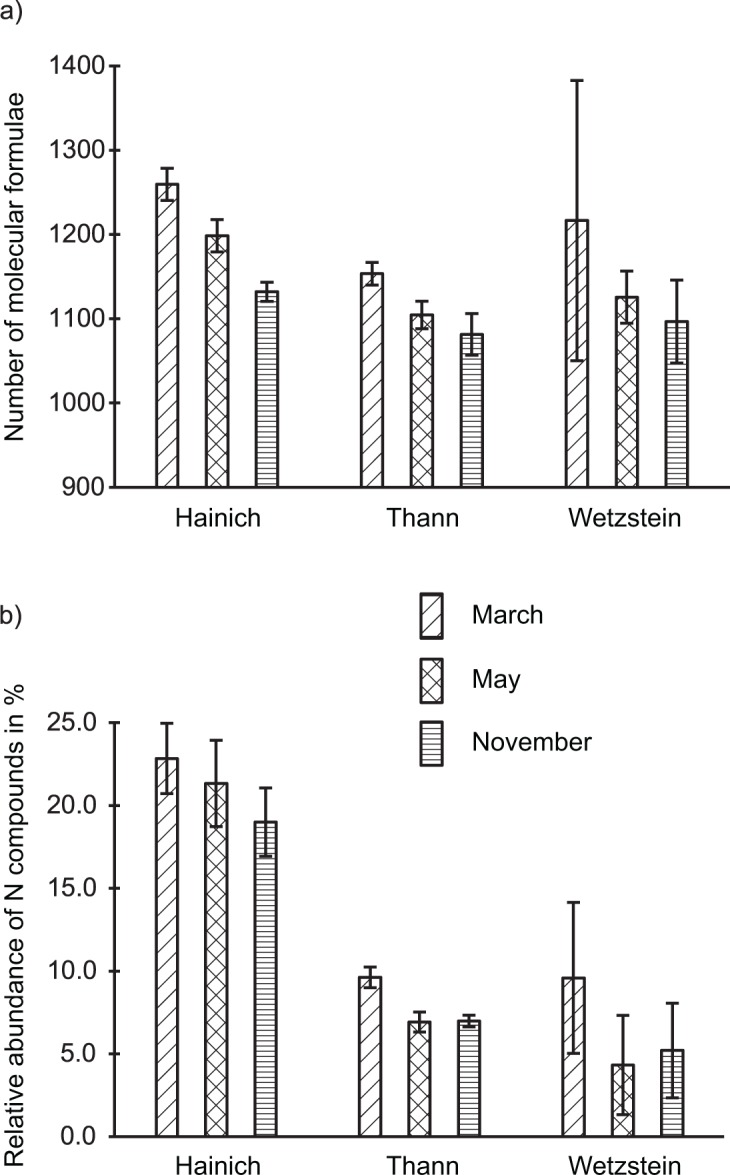
Overview of abundance of molecular formulae per month and main site. (a) Average abundance of formulae, (b) average relative abundance of nitrogen-containing compounds. Error bars indicate standard deviation between individual sampling spots or replicate measurements.

There was a clear trend of decreasing molecular diversity during the growing season from March to May and November for all main sites (Fig. 6a) which is consistent with the RDA results (Fig. 2). Additionally, the number of nitrogen-containing compounds decreased in the course of the year at all three sites, although the total number of nitrogen-containing compounds varied among the sites, with the highest number appearing at the Hainich site (Fig. 6b).

For a more detailed interpretation of these molecular trends, we identified those molecular formulae that were significantly correlated with the length of the growing season. This was based on the partial RDA within the variation partitioning between pH and DOC and the length of the growing season. Only four formulae fulfilled the criterion of R^2^ > 0.5 and they were all negatively correlated with the length of the growing season. Because so few formulae were found, they are not focus of the discussion.

**Fig 7:**
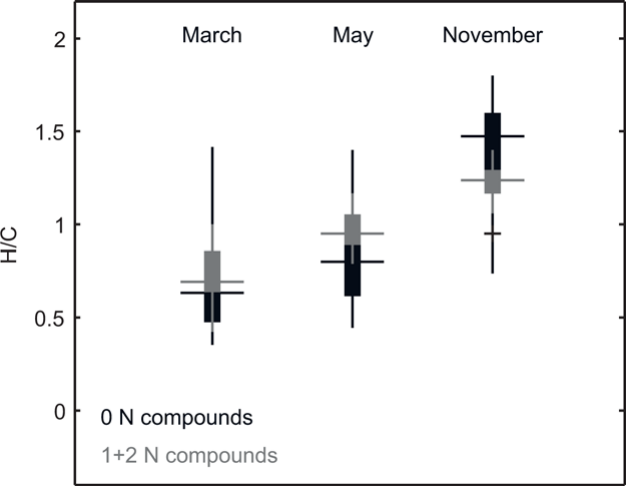
H/C data for unique molecular formulae for March, May and November subdivided into nitrogen-free and nitrogen-containing formulae presented as boxplots.

As an alternative approach to analyze seasonal trends on a molecular level, we identified those molecular formulae that occurred uniquely in each month. The resulting numbers of unique formulae were 105 for March, 33 for May and 140 for November. The main differences between monthly unique peaks were in H/C (Fig. 7), both for nitrogen-containing and nitrogen-free compounds. This increase in saturation over the growing season was more pronounced for nitrogen-free compounds compared to nitrogen-containing compounds.

### Comparison with the Yenisei

**Fig 8:**
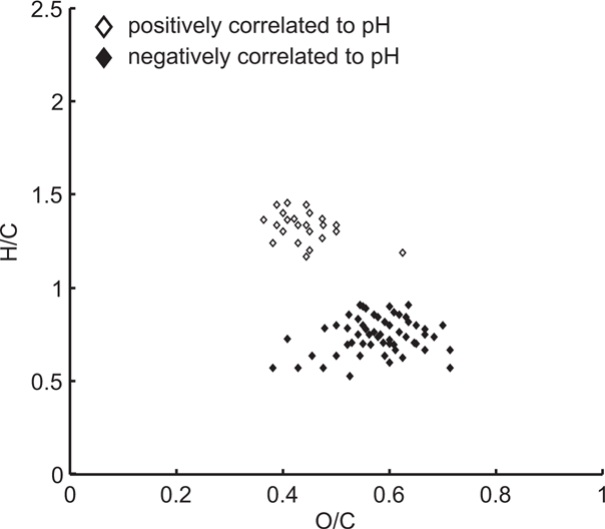
Van Krevelen diagram of those formulae that correlated with pH for both the Yenisei transect study and the forest soil water study.

**Fig 9:**
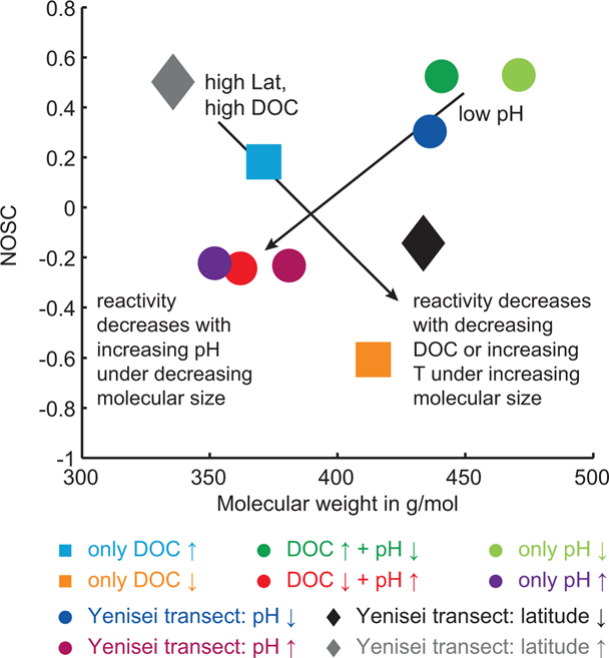
Overview of NOSC and molecular mass data of the different groups of molecular formulae that are significantly correlated with pH, DOC concentration and temperature from the Yenisei transect and forest soil water study. The data are summarized as centroid data.

We tested if the formulae that were significantly correlated with pH were the same in this study and the previous study in the Yenisei [29]. Both studies shared 23 molecular formulae that positively correlated with pH, and 57 that negatively correlated with pH. Consequently, 15% of the formulae that correlated positively with pH in this study and 22% of those in the Yenisei transect study were identical. In the case of formulae that were negatively correlated, 31% of those in this study and 26% of those in the Yenisei transect study were identical. Higher molecular saturation (H/C) was clearly related to higher pH (Fig. 8). In addition, we found in both studies that the molecular weight of the formulae was highest at high DOC concentration, low pH, and high temperature (Fig. 9). There was also a clear trend in NOSC, which was highest at high DOC concentration, low pH and low temperature (Fig. 9).

## Discussion

Knowledge of the factors that control DOM molecular composition is scarce. Here we tested the hypothesis that there is a universal relationship between DOM molecular composition and pH and temperature across different aquatic systems. We studied the differences in the molecular characteristics of forest soil water DOM. The pH variation was achieved by selecting three different forest sites in Germany that differ in soil pH. The temperature effect was separated by analyzing samples from three different seasons. Similar to a previous study in the Yenisei tributaries, we observed a statistical correlation between pH and DOM molecular composition. We demonstrated that pH, DOC concentration and the length of the growing season together explained 42% of the variability in the DOM composition. Even within a small pH range between pH 4.0 and 5.5, the molecular composition of DOM significantly correlated with pH and DOC concentration. DOC concentration and pH had a shared effect whereas the influence of the length of the growing season was independent of them.

As with any analytical method, FT-ICR-MS does not have an unlimited analytical window. This analytical window is very broad for ESI-FT-ICR-MS in the case of polar water-soluble organic compounds; however, small ionic compounds and colloidal matter are not likely to be detectable via this technique. This restriction must be kept in mind when interpreting our data.

### Trends driven by pH and DOC concentration

Correlations do not prove causal effects and in this study we cannot *a priori* exclude a correlating influence of pH and vegetation on the DOM molecular composition because different tree species grow at the three forest sites. The consistent pH trend observed in both the Yenisei tributaries and German forest soils, however, provides strong support for our hypothesis that pH is a main driver for DOM molecular composition. Consistently, a significant percentage of the formulae that correlated with pH were identical in both studies. Even if this does not prove that they originate from identical molecular structures, they at least have similar molecular properties, e.g., in terms of NOSC, saturation and molecular weight. This observation is consistent with findings from Strobel et al. [40]. They demonstrated that the chemical composition of the forest floor DOM of an equivalent DOC concentration does not depend on the tree species or soil type.

Higher DOC concentrations and lower pH were related to greater molecular unsaturation, greater oxygenation and larger molecular size. These characteristics indicate a higher abundance of unsaturated C = C and C = O bonds, an increased occurrence of oxygen functionalities and greater molecular size at a lower pH. These molecular characteristics and the corresponding distribution patterns in the van Krevelen diagram (Fig. 4) indicate that phenolic tannin compounds may be more abundant at a lower pH [30], [57]. Tannins are produced as secondary metabolites and are known to be produced at a higher rate when plants grow under environmental stress such as nutrient-poor soil, low pH or drought conditions [58–60].

In addition to the above characteristics, we observed a higher abundance of nitrogen-containing compounds at a high pH and low DOC concentration. The structure of these compounds remains unknown. One likely possibility is amino groups, which can be the predominant nitrogen-containing class in DOM and the soil organic matter of forest soils [61], [62]. Amino bonds occur, e.g., in amino sugars and peptides that are largely products of microheterotrophs and are assumed to be indicators of microbial activity [38]. The occurrence of tannins or similar polyphenols can reduce organic matter decomposition rates because tannins can precipitate and immobilize organic nitrogen compounds [14], [63], [64]. This mechanism may explain the lower diversity of nitrogen compounds at Wetzstein and Thann, the low pH sites. The greater diversity of nitrogen-containing compounds at the Hainich site may be the result of higher biological activity that could be related to lower tannin content and more neutral pH conditions. Additionally, the low MW at a higher pH may also be related to enhanced microbial activity. Processing the plant-derived material of high MW results in the release of compounds of lower MW and the buildup of the microbial biomass [65], [66]. Future studies at sites with different soil pH but the same vegetation and vice versa are needed to unambiguously separate the importance of the “nature” of DOM (the sources control DOM quality and reactivity) and the “nurture” that assumes that environmental variables drive DOM quality and reactivity [14], [26], [67], [68].

### Seasonal differences in DOM composition

In addition to the correlation of pH and DOC concentration with DOM molecular composition, RDA and variation partitioning demonstrated the seasonal dependency of the DOM molecular composition. The alignment along the PC3 axis according to the sampling month also indicated a seasonal influence. Both unconstrained and constrained ordination demonstrated that the greatest seasonal difference in molecular composition occurred between March and November (Figs. 1 and 2). The seasonal differences are related to the diversity of molecular compounds and the occurrence of nitrogen compounds (Figs. 2 and 6). There is a distinct trend of a greater number of compounds in March, whereas the number decreases into May and November. To explain this observation, we suggest the temperature dependence of microbial degradation coupled with little microbial degradation over winter, which may lead to the seasonal accumulation of compounds that are only slowly processed in winter. This supports recent findings suggesting that plant-derived compounds are only present when microbial activity and the decomposition rate are low [69]. During winter, the mineralization of nitrogen compounds is likely inhibited, which would explain the higher amount of these compounds present in March (Fig. 6). With higher temperatures, the biodegradation rate increases, possibly leading to a less diverse spectrum of compounds and to a greater extent of mineralization of nitrogen-containing compounds such as peptides or amino sugars [38], [62]. These conclusions are supported by our analysis of formulae unique to each month. Because the DOM of microbial origin is characterized by a low content of aromatic functionalities, the greater unsaturation in March indicates plant-derived (polyphenolic) molecules with a low degree of degradation [69], [70]. Due to rising temperatures over the course of year, microbial degradation increases, and may explain greater saturation. A similar trend was also observed for nitrogen-containing compounds but was less affected by increasing saturation compared to nitrogen-free compounds (Fig. 7). This may suggest a lower degree of degradation and higher rate of recycling, which supports other findings of preserved nitrogen-containing compounds during soil decomposition and humification, whereas organic compounds based only on C, H and O undergo increased degradation in their role as an energetic base [71], [72].

In summary, we discerned two aspects of seasonal differences across all sites: over the course of the growing season, the molecular diversity of DOM decreased and the molecular saturation increased. In the previous Yenisei study, the latitudinal gradient was correlated with the mean annual temperature and was considered as a space-for-time substitution to predict future climate change [29]. We suggested that the latitudinal variations were derived from temperature-dependent decomposition of DOM. Consequently, we concluded that increasing temperatures in the higher latitudes might increase DOM decomposition. Also in this study on soil DOM, we suggested the seasonal differences to be derived from temperature dependent degradation processes. In both studies, the DOM characteristics indicate substances with a lower degree of degradation occur in samples that are under the influence of lower temperature, i.e., samples taken at March at the beginning of the growing season and samples taken at higher latitudes. In addition, we demonstrated in both studies that the effect of pH on DOM composition was independent from a temperature influence, i.e., different compounds were affected by variations in pH and temperature.

## Conclusions

DOC concentration and pH are strongly correlated with DOM molecular composition. Because of the covariance of both parameters, their individual relationship to DOM composition was discerned for a limited number of molecular formulae. To fully separate the influence of DOC concentration, pH, vegetation and other parameters, future studies should consider a larger number of samples along independent pH and DOC concentration gradients. In addition, future studies should address a wider range of different environments. Our study design did not explicitly allow the identification of vegetation influences on DOM composition. However, an astonishing similarity of results from a previous study in Yenisei tributaries and this study in German soil pore waters is strong evidence that the identified pH dependence is universal. Even on the small pH range in the soils, the relationship between pH and DOM molecular composition became evident. We propose reduced microbial activity at low pH as the reason for the observed trends. We also observed a higher abundance of polyphenols (such as tannin) at low pH that may be vascular plant-derived. These compounds may adversely affect microbial metabolism and lead to the immobilization of nitrogen-containing compounds, which in turn would explain the observed distribution pattern of nitrogen compounds.

We found a seasonal trend in DOM composition in the soils that was consistent with the latitudinal trend in the Yenisei study. In particular, the total number of DOM compounds and the molecular unsaturation decreased in the course of the growing season in the soils, and from North to South in the Yenisei tributaries. We propose that microbial activity was slowed down during colder periods, causing an accumulation of compounds during winter. This accumulation could explain the greater molecular diversity of DOM. On a structural level, this is expressed by a higher abundance of aromatic and unsaturated compounds from plant-derived material in March (and in the North of the Yenisei), changing to more saturated material of microbial origin in May and November (and in the South of the Yenisei). The oxidation state of DOM (NOSC) also varied systematically along DOC, pH and temperature gradients, indicating that DOM is potentially more reactive during colder periods and in regions of low pH. This supports our conclusion that low temperature and low pH cause reduced biological degradation of DOM.

Overall, the findings support our hypothesis that abiotic factor such as pH and temperature dominate the molecular composition of DOM across biomes and different types of aquatic systems. Together with our recent Yenisei transect study, this indicates that basic environmental parameters, such as pH and temperature might be key controlling factors in the carbon cycle.

## Supporting Information

S1 Fig Political map of Germany with the geographical positions of the three sites Hainich, Thann and Wetzstein. (EPS)

S2 Fig Overview of mean daily temperatures and daily precipitation for the three sites Hainich, Thann (data for weather station at Weiden from ‘Deutscher Wetterdienst’: http://www.dwd.de.) and Wetzstein. The sampling periods for soil water collection (dates in Table 1) are highlighted with grey bars.
(EPS)

S1 Table List of measured mass, exact mass, number of C, H, N, O and S atoms, and relative intensities for each measurement (XLSX)

S2 Table List of measured mass, exact mass, number of C, H, N, O and S atoms, and relative intensities for each measurement of deep sea DOM reference sample replicate measurements that were used to describe peak reproducibility. The determined limit of detection for the group of four samples (LOD_Group_) was 7.90 * 10^-5^ and the limit of quantification for the group of four samples was set to 2 * LOD_Group_. All peaks with a relative intensity < 2 * LOD_Group_ were set to 1.5 * LOD_Group_ (1.19 * 10^-4^).
(XLSX)

S3 Table Molecular formulae information for ‘only DOC ↑’: List of measured mass, exact mass, number of C, H, N, O and S atoms, double bond equivalents (DBE) and aromaticity index (AI, [53]). (XLSX)

S4 Table Molecular formulae information for ‘only DOC ↓’: List of measured mass, exact mass, number of C, H, N, O and S atoms, double bond equivalents (DBE) and aromaticity index (AI, [53]). (XLSX)

S5 Table Molecular formulae information for ‘DOC ↑ + pH ↓’: List of measured mass, exact mass, number of C, H, N, O and S atoms, double bond equivalents (DBE) and aromaticity index (AI, [53]). (XLSX)

S6 Table Molecular formulae information for ‘DOC ↓ + pH ↑’: List of measured mass, exact mass, number of C, H, N, O and S atoms, double bond equivalents (DBE) and aromaticity index (AI, [53]). (XLSX)

S7 Table Molecular formulae information for ‘only pH ↓’: List of measured mass, exact mass, number of C, H, N, O and S atoms, double bond equivalents (DBE) and aromaticity index (AI, [53]). (XLSX)

S8 Table Molecular formulae information for ‘only pH ↑’: List of measured mass, exact mass, number of C, H, N, O and S atoms, double bond equivalents (DBE) and aromaticity index (AI, [53]). (XLSX)
